# Improving the properties of straw biomass rattan by corn starch

**DOI:** 10.1080/21655979.2019.1688127

**Published:** 2019-11-22

**Authors:** Yifan Dai, Yue Qiu, Junyang Jin, Qi Jia, Surendra Sarsaiya, Zhihao Wang, Wang Xin, Jishuang Chen

**Affiliations:** a College of Biotechnology and Pharmaceutical Engineering, Nanjing Tech University, Nanjing, Jiangsu, China; b Bioresource Institute for Healthy Utilization (BIHU), Zunyi Medical University, Zunyi, Guizhou, China

**Keywords:** Corn starch, straw biomass rattan, mechanical properties, melting performance, hydroscopicity, microstructures

## Abstract

As a kind of renewable resource and natural biomass, starch has been widely used to substitute plastics in the modern industry and is regarded as one of the most promising biodegradable materials. The newly developmental rattan, straw biomass rattan (SBR) as weaving material, has been exploited as per our previous work, which possessed advantages of both natural rattan and pure plastic rattan. The main objective of the work was to improve the properties of SBR by corn starch (CS). Based on the manufacturing of the above composites, the experiments of SBR that enhanced with CS on mechanical properties, melting performance, hydroscopicity, thermogravimetric analysis, and microstructures were tested in this study. The results revealed that when the content of CS increased gradually within the range of 0, 3, 6, 9 12, and 15 wt.%, the mechanical properties and melt index of the composite both increased first and then decreased, with 6 to 12 wt.% as the optimal dosage range. In contrast, the water absorption of SBR kept increased in this range, indicating an easier biodegradable. With CS added, the microstructure of SBR was examined by scanning electron microscope and found the microscopic surfaces and sections to become smoother, and that could improve the compatibility and tenacity between the materials. As a result, CS in moderation can be used as a supplement to enhance SBR, and improve their characteristics which will enhance the mechanical properties of the composites for future perspectives.

## Graphical abstract

**Figure:**
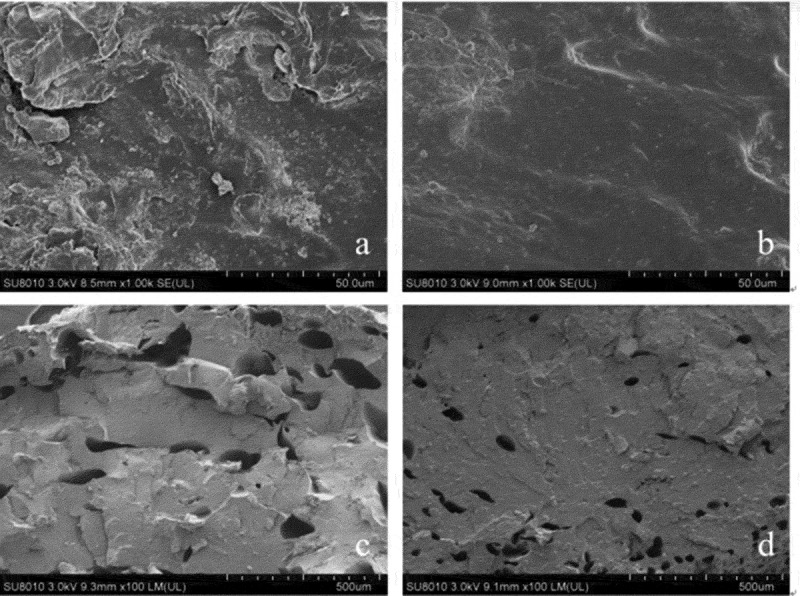

## 1. Introduction

In modern rattan weaving industry, the natural rattan and pure plastic rattan are the main weaving materials. But in the use process of these materials, the negative effects were occurred frequently [1]. Natural rattan cannot be produced in high quantity, its price was increased invariably due to it was mainly produced in southeast Asia and these countries limited the export of natural rattan [2]. Moreover, it was considerably easier to become fading, embrittlement, strength reduction and damage by insects. Generally, weaving industry chooses to immerse the rattan in paint or liquid reagent to alleviate this problem. These approaches violate the concept of environmental protection and neither economical nor healthy. Based on this situation, pure plastic rattan has occupied more and more market share with its favorable anti-insect, anti-mildew effect, stable quality and mass production [3]. However, the white pollution caused by the use of plastic makes it difficult to establish a foothold in the high-end furniture industry which makes to find new alternatives becomes necessary.

As new environmental protection material, straw plastic composites (SPC) was produced by a special process which based on utilize modification technology to increase the interfacial compatibility between straw fiber and polymer [4]. It had been paid great attention and was widely used in plentiful areas such as daily necessities, furniture industry, municipal engineering, automobile industry and the like [5]. Actually, the raw materials of SPC are not limited to straw and add in different biomass will give SPC diverse functional characteristics [6]. For instance, the addition of wormwood powder or coffee grounds will make the composites with fragrance and could let it has been improvements in deworming and refreshing. As the combination of SPC and pure plastic rattan, straw biomass rattan (SBR) has already created by us which has the advantages of both natural rattan and pure plastic rattan. It can not only be made in quantity production and variety of shapes, but also with benign tactility and excellent mechanical properties. Furthermore, it is able to satisfy the weaving requirements after practical experiences by manufacturers and has already applied to chair, table, handwork, packaging and so on.

At present, the straw leads to severe environmental pollution and resource waste [7]. Its processing methods include chemical, physical, and biological methods [8–10]. Straw fibers, which have strong polarity due to their unique structure and composition [11]. On the contrary, the polyethylene matrix is considered to a nonpolar polymer because it has no polar groups on the side chain and exerts fortissimo hydrophobic [12]. Based on the above, straw fibers have poor compatibility with the polyethylene. Therefore, the modification between the materials in SPC process is necessary. Microscopically, this step could reduce the polarity of the straw fiber and improve the interfacial strength between fiber and polyethylene matrix. From a macroperspective, the mechanical properties of the composites could be increased within limits with the improvement in compatibility of the fiber–polymer interface [13].

Besides wheat and corn grains, there are abundant starchy feedstocks, such as wasted crop, cereal bran, and potato peels [14]. Starch, as a natural polymer and can be completely biodegradable is supposed to have great potential to substitute plastic [15]. However, the decomposition temperature of starch is lower than the melting point which results in the material almost have no plasticity [16]. Besides, another factor that limits the application of starch is its strong water absorption which will accelerate the aging of materials in mechanical property [17]. The main aim of this research was to obtain the effects of CS addition on the properties of SBR. The above effects in this article include mechanical properties, melting performance, hydroscopicity, thermal stability, and compatibility in microstructures.

## 2. Materials and methods

### 2.1. Materials

Wheat straw (WS), size about 40 to 60 meshes, was collected in Shenqiu County (Zhoukou city, Henan province, China). Linear low-density polyethylene (LLDPE, DFDA-7042, Sinopec Yangzi Petrochemical Co., Ltd. China) was the matrix material of SBR. The filler was talc powder (1250 meshes, Suzhou Mingjiang Special Chemicals Co., Ltd. China) and used to enhance the rigidity of composite. Maleic anhydride grafted polyethylene (MAH-g-PE, Polymirae Co., Ltd., Korea) was used as the modifying agent to promote fusion between straw and LLDPE. Similarly, the silane coupling agent (KH-550, Sinopec Yangzi Petrochemical Co., Ltd. China) was to change the compatibility between talc powder and LLDPE. Corn starch (CS, Qinhuangdao Lihua Starch Co., Ltd.), which was almost the size of about 800 to 1250 meshes used as an intensifier in this study.

### 2.2. Preparation of the materials

First of all, the WS and CS were placed in drying oven (GZX-9246, Shanghai Boxun Medical biological instrument Co., Ltd. China) under 80°C for 24 h to ensure that its moisture content below 5% to prevent foaming phenomenon in the materials. The moisture content was determined by using moisture analyzer (MA35M-000230V1, Beijing Sartorius Scientific Instruments Co., Ltd. China) and the parameter setting as following: the setting temperature was 105°C while the time period was 60 min. The modification of WS and talc powder was initiated in a high-speed mixer (SHR-50A, Zhangjiagang Gelan Machinery Manufacturing Co., Ltd. China). 75 wt.% WS was homogeneously mixed in the mixer with 20 wt.% talc powder and 5 wt.% silane coupling agent under the set temperature of 120°C for at least 10 min. At the end of this process, the modified powder was obtained.

### 2.3. Product fabrication

Primarily, the production of SBR needs to be pelletted to guarantee the experiment can be conducted smoothly. A varying weight fraction (0, 3, 6, 9, 12, and 15 wt.%) of CS was blended with (85, 82, 79, 76, 73 and 70 wt.%) pure LLDPE, respectively. Then, mix the blend with 5 wt.% MAH-g-PE and 10 wt.% modified powder by using a laboratory pan at indoor temperature. Afterward, the mixture was poured into atwin-screw extruder (T20, Nanjing Kebeilong Machinery Manufacturing Co., Ltd. China) to acquire biomass granule and its parameter setting as following in Table 1.

**Table 1.** Technological condition of granulation.

Parameter name	Parameter setting
I area temperature	160°C
II area temperature	160°C
III area temperature	160°C
IV area temperature	170°C
The outlet temperature	180°C
Main engine rate	400 r/min

SBR was extruded by add biomass granule into a single-screw extruder (Φ55 & Φ30*2, Yaoan Plastic Machinery Co., Ltd. China) and its parameter setting as following in Table 2. The extrusion tool of this machine was four-wire rattan with a width of 10 mm and a thickness of 1 mm.

**Table 2.** Technological condition of extruding.

Parameter name	Parameter setting
I area temperature	160°C
II area temperature	160°C
III area temperature	160°C
IV area temperature	170°C
The outlet temperature	180°C
Main engine rate	400 r/min
Feeding rate	40 r/min
Traction driver frequency	3.0
Winding driver frequency	1.5

### 2.4. Property test

#### 2.4.1. Mechanical property analysis

The mechanical properties about SBR in this paper include the tensile strength and elongation at break which could reflect the actual carrying capacity and elastic property. The measuring method of these two indexes was conducted by ASTM D 882–02 (standard test method for tensile properties of thin plastic sheeting) and determined with a universal testing machine (UTM-1422, Chengde Jinjian Testing Instrument Co., Ltd. China) [18]. The test conditions were set as follows: the length of each sample was 36.5 cm while the rate of extension was 50 mm/min and the standard spacing of the sample was 50 mm. The set fracture factor was 0.5. There were five test samples for each formula.

#### 2.4.2. Melting performance analysis

The melt index was indicated the fluidity of plastic materials during processing while the melting performance of disparate CS content in SBR was tested by melt flow rate meter (FMI-1221, Chengde Jinjian Testing Instrument Co., Ltd. China) with ASTM D 1238-04a (standard test method for melt flow rates of thermoplastics by extrusion plastometer) [19]. The experimental parameters of the machine were set as follows: the test temperature was 190°C, while the test load was 5 kg. The times of shear for each sample was 5, while the shear time interval was 10 s.

#### 2.4.3. Hydroscopicity analysis

In this test, the hydroscopicity of SBR in 7-day was measured by following ASTM D 570–98: standard test methods for water absorption of plastics [20]. First, every representative specimen was processed to a size of 60 mm × 60 mm × 1 mm and had five parallels for each specimen. Second, all of specimens were dried in the oven at 105°C for about 24 h and then removed to a desiccator before weighing by an electronic analytical balance (Shanghai Youke Instruments Co., Ltd. China) with the balance precision of 0.0001 g. Then, every specimen was immersed into a distilled water bath at 24°C for 168 h. At the end of the immersion period, the specimens were removed from the water and the water on the surface was wiped off with a clean dry towel prior to weighing them. Percentage increase in weight during immersion was calculated to the nearest 0.01% using the following formula:

$$W,\%=\left(\frac{W_{2}-W_{1}}{W_{1}}\right)\times 100\%$$

**Equation 1**. Percentage increase in weight during immersion. Note: W is water absorption. W_1_ is the conditioned weight of the specimen. W_2_ is the wet weight of the specimen.

### 2.5. Thermogravimetric analysis (TGA)

TGA is measuring the relationship between the mass of material and temperature. The thermal stability of the pure LLDPE, raw CS and SBR with none CS, 15 wt.% CS was assessed by synchro thermal analyzer (STA 449F3, Netzsch Scientific Instruments Trading Co., Ltd., Germany). The test was done in a nitrogen (N_2_) atmosphere under a flow rate of 60 mL/min to prevent oxidation. Approximately 20 mg of each sample was placed on a platinum pan and heated from ambient temperature to 600°C at the heating rate of 10°C/min.

### 2.6. Scanning electron microscopy (SEM)

SEM of the SBR with none CS and 15 wt.% CS was used to evaluate the consistency between materials. In this observed test, the surfaces and sections of the SBR were examined with a scanning electron microscope (SU8010, Hitachi Manufacturing Co., Ltd., Japan). In addition, the reason about enhancement of material mechanical properties by CS in the polymer matrix was discussed. The selected specimens were frozen in liquid N_2_ for 60 s before fracture it, and then observed the sections of it. Afterward, the processed specimens were mounted on a copper plate with a black sticky band and sputtered it with gold prior to take microscopic analysis.

## 3. Results and discussion

### 3.1. Results of mechanical properties

#### 3.1.1. Tensile strength

**Figure 1.**
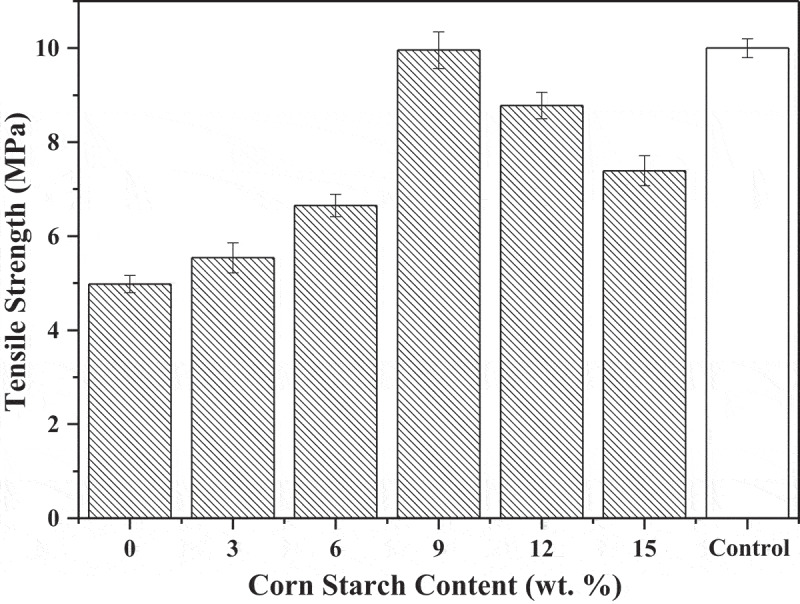
Tensile strength versus CS content of SBR.

As shown in Figure 1, the tensile strength of varying CS content on SBR is summarized. The results show that with the CS content increased, this index increased up firstly and then turn decreased. When the CS content reaches to 9 wt.%, the tensile strength of SBR keeps at a high level and reaches to a maximum of 9.96 MPa which resembles pure LLDPE rattan. When CS reaches an appropriate proportion, the blend has a preferable binding force with polymer, which forms stable blends and improves their mutual affinity and compatibility. The principal reason for the tensile strength increased could be that the CS acted as ‘plasticizer’ when it dispersed in the LLDPE matrix. Therefore, the composite ultimately exhibits a benign mechanical property. With the CS was added in excess of 9 wt.%, the tensile strength decreased gradually. This result could be due to the presence of excessive CS, and redundant CS particles agglomerated into clusters and cohered in stress concentration points. Meanwhile, CS had poor dispersion in LLDPE matrix which greatly influenced the continuity of rattan. Eventually, the composite might undergo fracture in this stress concentration point when an external force is applied.

#### 3.1.2. Elongation at break

**Figure 2.**
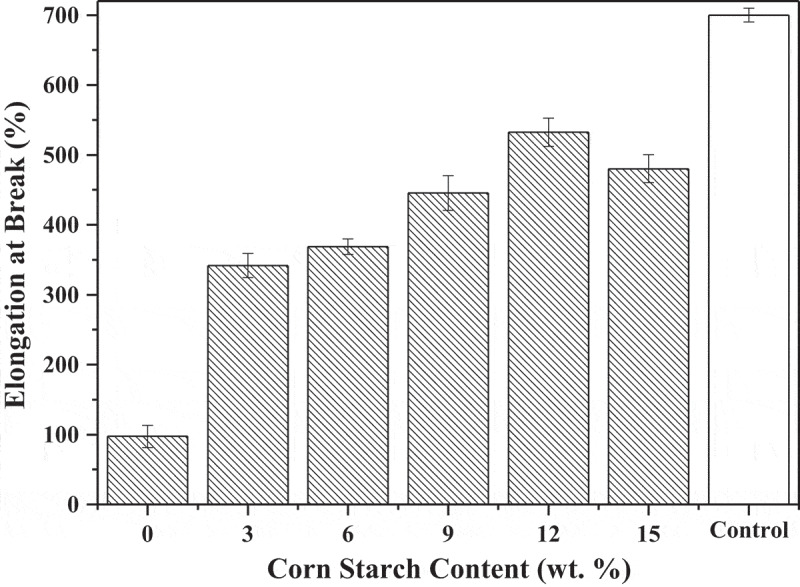
Elongation at break versus CS content of SBR.

Elongation at break versus CS content of SBR is shown in Figure 2, the value is first increased and then slightly decreased. The peak value of elongation at break reaches 532.44%, while the CS content accounted for 12 wt.%. However, it is still inferior to LLDPE which reaches about 700%. With CS addition, this indicator reveals a great difference. A small amount of CS could enhance greatly elongation at break in SBR. The principal reason for this phenomenon could be the grain diameter of CS is 800 to 1250 meshes which size is small and easier to disperse in the blend, then enhanced the continuous phase of the composite. When the specimens were continuously under stress, each rattan underwent elastic deformation and was not able to return to the previous shape. Consistent with tensile strength, elongation at break of the composite decreased in the end due to CS progressively increased which was conglomerated.

Two aspects in the mechanical properties of the rattan have a similar difference dynamic trend. To summarize, tensile strength and elongation at break of the rattan were at high levels when the CS content was 9 wt.% and 12 wt. %, respectively.

### 3.2. Melting performance

**Figure 3.**
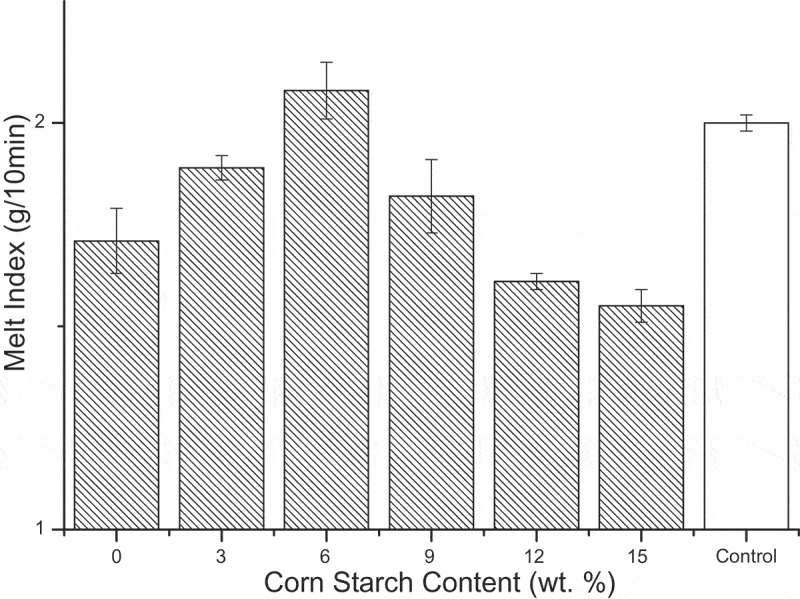
Melting performance versus CS content of SBR.

The melting performance of CS as reinforcement in SBR is presented in Figure 3. With the increase of CS, the melting index of SBR increased obviously and after more than 6 wt.%, it shows a downward trend. In a certain range, CS due to the small particle size and serve as lubricant had preferable dispersion in the LLDPE matrix which could enhance the flow performance of the SBR in order to reduce the loss of the machine [21]. As the CS content further increased, starch was agglutination together and even exposed to the surface of the blend, thus causing plugging in the material which makes its melting performance terrible. In brief, the composite shows a great melting performance when CS content reached to 6 wt.% and even a little more than pure LLDPE.

### 3.3. Hydroscopicity

**Figure 4.**
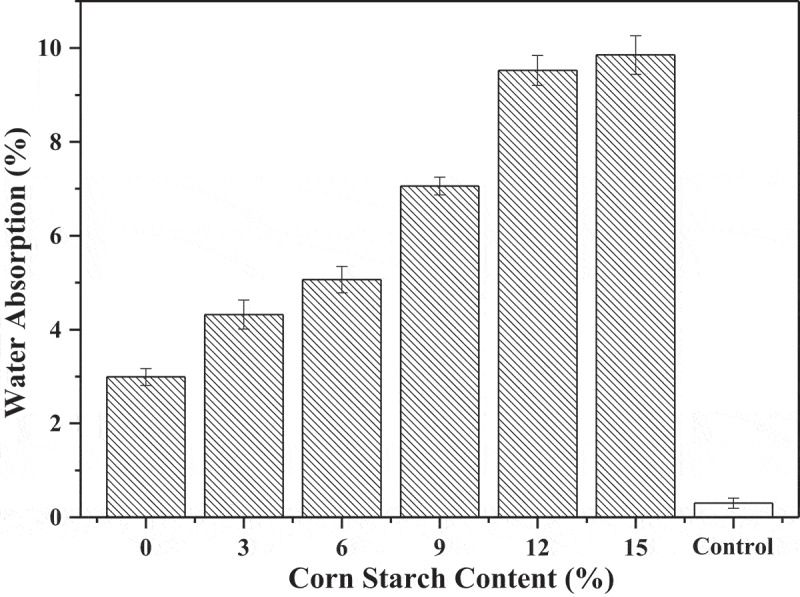
Hydroscopicity versus CS content of SBR.

In Figure 4, the effect of CS on 7-day water absorbency of SBR has been demonstrated. Overall, the higher CS content in the rattan, the higher the SBR water absorption. This can be explained as a higher moisture absorption and more hydrophilic tendency of the starch, part of which after being made into SBR was exposed to the air rather than wrapped in the LLDPE matrix [22]. On the surface of the composite, CS was not wrapped with polymer and direct contact with water which result in the composite material with starch has poor water resistance. In the employ of composites, their waterproofness played an important role which will not only affect the mechanical properties of the composite but also affect the service life of them [23]. However, it enhanced the biodegradability of SBR in use [24].

### 3.4. Thermogravimetric analysis (TGA)

**Figure 5.**
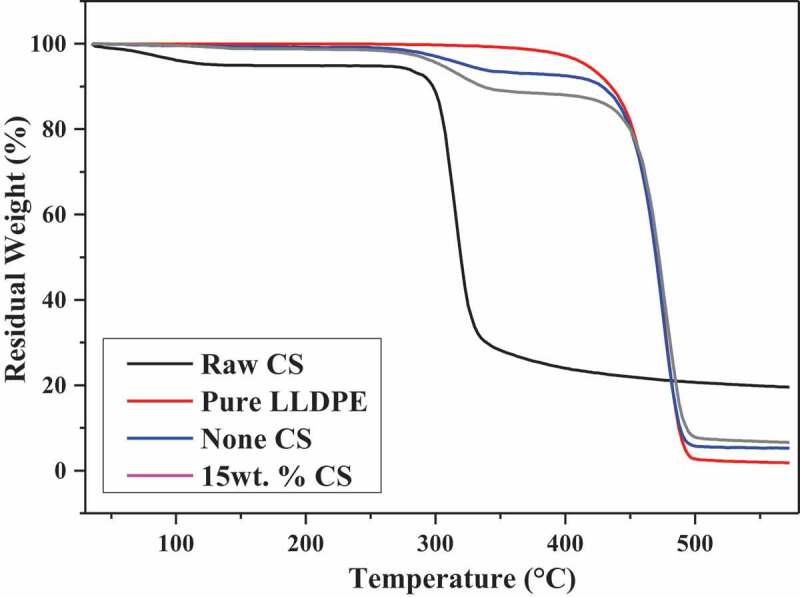
TGA versus CS content of SBR.

**Figure 6.**
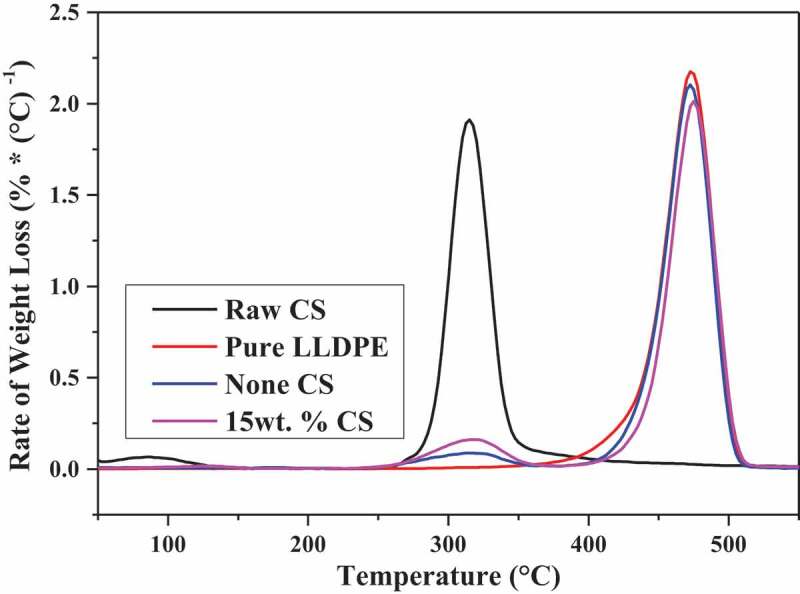
DTG versus CS content of SBR.

The thermogravimetric analysis was conducted to determine the thermal stability of pure LLDPE, raw CS and reinforced SBR with none CS and 15 wt.% CS. The TGA and DTG (differential thermal gravity) reading curves of the above materials are shown in Figures 5 and 6, respectively. Weight loss in the thermal degradation process of CS occurred in two successive phases. The initial weight loss of the starch was occurred in the temperature range of 35°C to 105°C due to the evaporation of moisture. The eventual weight loss (280°C to 350°C) was associated with the oxidation degradation of starch molecules [25]. For pure LLDPE, the weight loss of it has only one phase and proceed in around 410°C to 500°C. Because of the addition of biomass, the thermostability of composites has changed remarkably but both worse than pure LLDPE. On the basis of DTG graphs, the maximum decomposition rate of CS is at 310°C while the other materials are both at around 470°C. With the addition of biomass, the thermal degradation onset temperature of overall composite materials was in advance to around 310°C which signified the composite presented lower thermal stability than pure LLDPE.

### 3.5. Scanning electron microscopy (SEM)

**Figure 7.**
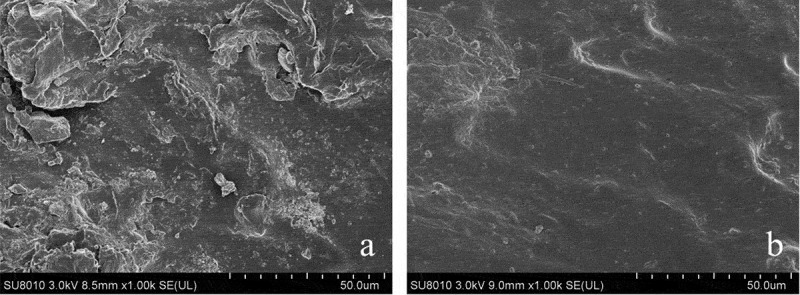
The surfaces of SBR with none (a) and 15 wt.% (b) CS content.

Examination of surfaces about the rattan with none and 15 wt.% CS content is shown in Figure 7. These SEM images express that with the addition of CS, the surface of SBR appeared smoother in microstructure. This can be interpreted as CS increased the texture of SBR and the compatibility between materials.

**Figure 8.**
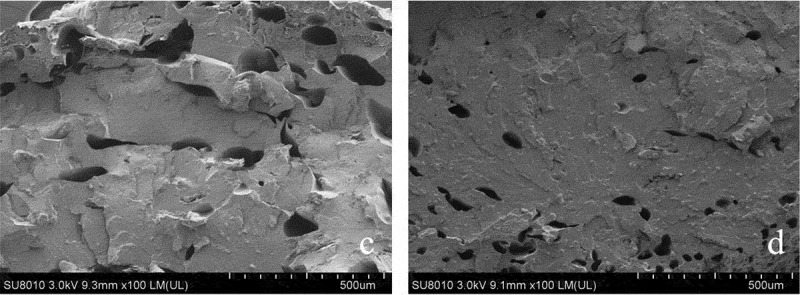
The sections of SBR with none (c) and 15 wt.% (d) CS content.

The micrographs in Figure 8 show the structural difference in the sections of SBR between before and after CS addition. Obviously, the section appearance of the composites is significantly changed. The section of SBR with none CS was emerged larger porosity than another specimen. This can be attributed to CS increased the continuity of SBR. These results are consistent with the mechanical properties of the composites above.

## 4. Conclusions

The mechanical properties of SBR in this paper mainly refer to tensile strength and elongation at break which values both increased up firstly and then turned decreased. The elongation at break of SBR was greatly affected by the addition of CS. Only added 3 wt.% CS could make this index increase triple. When the CS content was 9 wt.% and 12 wt.%, tensile strength and elongation at break of the composites were at high levels, respectively. The melting performance of SBR had a similar dynamic trend with mechanical properties and the optimal number content for this index was 6 wt.%. Based on starch is hydrophilic, the water absorption of the SBR was still rose with the CS increased which indicated the good degradation of it. The principal reason could be that the starch was exposed to the surface of the composites and directly contact with air rather than wrapped in the LLDPE matrix.

The TGA analysis revealed that CS had a relatively lower thermal degradation temperature (around 280°C to 350°C) than pure LLDPE (around 410°C to 500°C). Moreover, the thermal stability of SBR was reduced slightly with the addition of biomass. The SEM micrograph analysis clearly demonstrated that the surfaces and section structure, respectively, changed remarkably following the addition of CS which can be attributed to increased compatibility and continuity between materials. Accordingly, this phenomenon could confirm the CS enhanced the mechanical properties of the composites.

## References

[1] Lu X, Liu Y, Ni Y, et al. Study on the folding of imitation rattan with wheat straw fibers. J Biobased MaterBioenergy. 2017;11(4):303–307.

[2] Myers R. What the Indonesian rattan export ban means for domestic and international markets, forests, and the livelihoods of rattan collectors. For Policy Econ. 2015;50:210–219.

[3] Chen Z, Zhang J, Chen H, et al. Analysis of design elements of plastic rattan furniture. Furniture Inter Des. 2012;2:18–19.

[4] Zhang W, Chen J. Development and industry prospect of Straw-Plastic Composites. Bull Sci Technol. 2016;1:97–101.

[5] Chen J, Liu Y. Industrial utilization of straw biomass and the straw plastic composition. J Jiangsu Normal Univ (Natural Science Edition). 2015;3:31–35.

[6] Bekele LD, Zhang W, Liu Y, et al. Impact of cotton stalk biomass weathering on the mechanical and thermal properties of cotton stalk flour/linear low density polyethylene (LLDPE) composites. J Biobased Mater Bioenergy. 2017;11(1):27–33.

[7] Wang Y, Shao Y, Zou X, et al. Synergistic action between extracellular products from white-rot fungus and cellulase significantly improves enzymatic hydrolysis. Bioengineered. 2017;9(1):178–185. 28384075 10.1080/21655979.2017.1308991 PMC5972936

[8] Zhang J, Hou H, Chen G, et al. The isolation and functional identification on producing cellulase of Pseudomonas mendocina. Bioengineered. 2016;7(5):382–391. 27710430 10.1080/21655979.2016.1227143 PMC5060976

[9] Sharma D, Garlapati VK, Goel G. Bioprocessing of wheat bran for the production of lignocellulolytic enzyme cocktail by Cotylidia pannosa under submerged conditions. Bioengineered. 2016;7(2):88–97. 26941214 10.1080/21655979.2016.1160190 PMC4879985

[10] Yanagisawa M, Kawai S, Murata K. Strategies for the production of high concentrations of bioethanol from seaweeds. Bioengineered. 2013;4(4):224–235. 23314751 10.4161/bioe.23396 PMC3728193

[11] Xie Y, Hill CAS, Xiao Z, et al. Silane coupling agents used for natural fiber/polymer composites: A review. Composites Part A. 2010;41(7):0–819.

[12] Jin DW, Seol SM, Kim GH. New compatibilizer for linear low-density polyethylene (LLDPE)/clay nanocomposites. J Appl Polym Sci. 2010;114(1):25–31.

[13] Zhang W, Chen J, Bekele LD. Physical and mechanical properties of modified wheat straw-filled polyethylene composites. Bioresources. 2016;11(2):4472–4484.

[14] Favaro L, Jooste T, Basaglia M, et al. Designing industrial yeasts for the consolidated bioprocessing of starchy biomass to ethanol. Bioengineered. 2013;4(2):97–102. 22989992 10.4161/bioe.22268 PMC3609629

[15] Siracusa V, Rocculi P, Romani S, et al. Biodegradable polymers for food packaging: a review. Trends Food SciTechnol. 2008;19(12):0–643.

[16] Liu H, Xie F, Yu L, et al. Thermal processing of starch-based polymers. Prog Polym Sci. 2009;34(12):1348–1368.

[17] Kiatkamjornwong S, Chomsaksakul W, Sonsuk M. Radiation modification of water absorption of cassava starch by acrylic acid/acrylamide. Radiat Phys Chem. 2000;59(4):413–427.

[18] ASTM D 880–02. (2002). ASTM International, Standard test method for tensile properties of thin plastic sheeting. West Conshohocken, Pennsylvania.

[19] ASTM D 1238-04a (2004). ASTM International, Standard test method for melt flow rates of thermoplastics by extrusion plastometer. West Conshohocken, Pennsylvania.

[20] ASTM D 570–98 (2010). ASTM International, Standard test methods for water absorption of plastics. West Conshohocken, Pennsylvania.

[21] Ogunjimi AT, Alebiowu G. Flow and consolidation properties of neem gum coprocessed with two pharmaceutical excipients. Powder Technol. 2013;246:187–192.

[22] Abral H, Dalimunthe MH, Hartono J, et al. Characterization of tapioca starch biopolymer composites reinforced with micro scale water hyacinth fibers. Starch - Starke. 2018;70:7–8.

[23] Kamisho T, Takeshita Y, Sakata S, et al. Water absorption of water-based anticorrosive coatings and its effect on mechanical property and adhesive performance. J Coat Technol Res. 2014;11(2):199–205.

[24] Yew GH, Yusof AMM, Ishak ZAM, et al. Water absorption and enzymatic degradation of poly(lactic acid)/rice starch composites. Polymer Degrad Stab. 2005;90(3):488–500.

[25] Zhou Y, Li X, Lv Y, et al. Effect of oxidation level on the inclusion capacity and solution stability of oxidized amylose in aqueous solution. Carbohydr Polym. 2016;138(1):41–48. PMID: 26794736 26794736 10.1016/j.carbpol.2015.11.040

